# PReS-FINAL-2083: The rate of malignancy in iranian children with joint manifestations

**DOI:** 10.1186/1546-0096-11-S2-P95

**Published:** 2013-12-05

**Authors:** N Shafaie, Y Aghighi, SR Raeeskarami, A Malek, MR Nesabi, A Izadpanah, B Nourbakhsh, SH Faghihi, Z Oloomi-Yazdi, P Vosough

**Affiliations:** 1 Iran Pediatric Rheumatology Center, Vali-e-Asr Hospital, Tehran University of Medical Sciences, Tehran, Iran, Islamic Republic Of; 2 Children's Hematology/Oncology Research Center, Ali Asghar Hospital, Iran University of Medical Sciences, Tehran, Iran, Islamic Republic Of

## Introduction

Different reports of shared manifestations for malignancy and rheumatic diseases in children since 1970s indicate the presentation of malignancies, particularly ALL, with musculoskeletal (MS) symptoms.

## Objectives

To evaluate the rate and the characteristics of hematological malignancies in Iranian children with joint manifestations based on two different scopes of data collection since 1981.

## Methods

In this retrospective review, we considered two different aspects of evaluation within two pediatric tertiary centers in Iran: 1- Children's Hematology/Oncology Research Center, 2- Pediatric Rheumatology Center.

## Results

Here we report the results of 3 studies (I, II & III) conducted through 1981-2006, followed by current (IV) study performed since 2006. Findings of four different studies are mentioned in detail, in Table 1. An additional study was performed in parallel to study I comprising 46 dead patients in addition to 54 current patients, which showed the similar results. ALL was the most frequent type of malignancy (80-100%).

**Table 1:** 

Period of Study	Study Center	Malignant cases with MS symptoms/total patients	%	Sex %	Mean age at diagnosis (years)	Mean delay to diagnosis (months)	Most common age group (years)	Most commonly involved Joints	Other Manifestations	Laboratory findings
(I) 1981-1995	Children's Hematology/Oncology Research Center	54/1528	3.5	M:62 F:38	7.3	NA	6-7	Knee 68% Ankle52%	Organomegaly 83% Lymphadenopathy 76%	Anemia 59% Leukopenia 18% Leukocytosis 61% Thrombocytopenia 55% Raised ESR 37%
	
(II) 1981-2003		76/1832	4.1	M:56.6 F:46.4	6.97	NA	5-7	Knee 55% Ankle48%	NA	Anemia 88% Leukopenia 34% Leukocytosis 14% Thrombocytopenia 72% Raised ESR 58% Raised LDH 34%

(III) 1996-2006	Pediatric Rheumatology Center	38/722	5.2	M:52 F:48	5.6	2.7	5-7	Hip 37% Knee 34%	Fever 73% Weight loss 60% Organomegaly 60%	Anemia 79% Leukopenia 45% Leukocytosis 16% Thrombocytopenia 63%
	
(IV) 2006-2012		11/207	5.3	M:73 F:27	6.6	3	6-7	Knee 45% Hip 27%	Fever 45% Organomegaly 27%	Anemia 27% Leukopenia 27% Leukocytosis 63% Thrombocytopenia 45%

## Conclusion

The relative increase in the rate of malignancy in this review (period of 1981-2012) could be an alarm to perform BM aspiration in children with joint manifestations harboring the following as "Red Flags": (i) Boys at the age of 5-7, (ii) Large joint involvement (particularly hip, knee and ankle) and (iii) Arthralgia at presentation. According to recent recommendations by ACR for early aggressive treatments in JIA patients, this precautionary measure may minimize the potential dangers and consequences of misdiagnosis prior to initiation of any treatment.

## Disclosure of interest

None declared.

